# Changes in subjective knee function and psychological status from preoperation to 6 months post anterior cruciate ligament reconstruction

**DOI:** 10.1186/s40634-022-00551-2

**Published:** 2022-12-01

**Authors:** Shunsuke Ohji, Junya Aizawa, Kenji Hirohata, Takehiro Ohmi, Sho Mitomo, Hideyuki Koga, Kazuyoshi Yagishita

**Affiliations:** 1 Clinical Center for Sports Medicine and Sports Dentistry, Tokyo Medical and Dental University, 1-5-45 Yushima, Bunkyo-Ku, Tokyo, 113-8519 Japan; 2 Department of Physical Therapy, Juntendo University, 3-2-12 Hongo, Bunkyo-Ku, Tokyo, 113-0033 Japan; 3 Department of Joint Surgery and Sports Medicine, Tokyo Medical and Dental University, 1-5-45 Yushima, Bunkyo-Ku, Tokyo, 113-8519 Japan

**Keywords:** Anterior cruciate ligament reconstruction, Fear of reinjury, Psychological recovery, Subjective outcome, Return to play

## Abstract

**Purpose:**

To determine characteristic changes in subjective knee function, kinesiophobia, and psychological readiness to return to sports between scores taken before anterior cruciate ligament reconstruction (ACLR) and those taken 6 months post-ACLR.

**Methods:**

Thirty-two participants (median age, 20.0 years) were included. Subjective knee function was assessed using the International Knee Documentation Committee Subjective Knee Form (IKDC-SKF). The Tampa Scale for Kinesiophobia (TSK-11) and Anterior Cruciate Ligament Return to Sport after Injury (ACL-RSI) scale were used to evaluate kinesiophobia and psychological readiness to return to sport, respectively. Questionnaires were administered 1 day before surgery and at 6 months post-ACLR. A positive change was defined as an increase in IKDC-SKF and ACL-RSI scores and a decrease in TSK-11 score. The change in each score from pre-ACLR to 6 months post-ACLR was analyzed using a paired t-test. The percentage change in scores was calculated, and the correlations of the percentage change in the TSK-11 and ACL-RSI scores and that in the IKDC-SKF score were analyzed.

**Results:**

All scores differed significantly positively from pre-ACLR to 6 months post-ACLR. The proportion of participants whose scores did not change positively from pre-ACLR to 6 months post-ACLR was higher for the TSK-11 (38.0%) and ACL-RSI (38.0%) than for the IKDC-SKF (6.3%). No correlation was observed between the percentage change in the IKDC-SKF score and that in the TSK-11 or ACL-RSI scores from pre-ACLR to 6 months post-ACLR.

**Conclusions:**

Changes in subjective knee function and psychological status from pre-ACLR and 6 months post-ACLR may not be interdependent.

## Background

Patient-reported outcome measures (PROMs) are widely used to assess knee function and psychological states of patients after anterior cruciate ligament (ACL) reconstruction (ACLR). The International Knee Documentation Committee Subjective Knee Form (IKDC-SKF) is a PROM used to evaluate the subjective knee function of patients after ACLR [34]. The Tampa Scale for Kinesiophobia (TSK-11) and the Anterior Cruciate Ligament Return to Sport after Injury (ACL-RSI) scale are PROMs used to evaluate the psychological state of patients after ACLR [22]. Poor scores on these measures are associated with poor outcomes, such as return to sport (RTS) failure and reinjury [14, 18, 25, 33].

Changes in PROM scores are typically evaluated at 6 months after ACLR. This is an important time point in postoperative rehabilitation because 6 months is the minimum period preceding the point when patients are allowed to start training for sports-related activities or participate in sports [7]. Therefore, assessing the changes in PROM scores from pre-ACLR to 6 months post-ACLR is important.

Several cohort studies have shown that IKDC-SKF, TSK-11, and ACL-RSI scores improved from pre-ACLR to 6 months post-ACLR [8, 17, 26, 28]. However, lack of improvement or worsening of scores at 6 months postoperatively is commonly observed in the clinical setting. Numerous patients have had increased TSK-11 scores and decreased ACL-RSI scores despite improved IKDC-SKF scores. Ohji et al. [24] showed that 30% (15/50) of ACLR patients had lower ACL-RSI scores from preoperation to 6 months postoperation. However, this previous study did not show IKDC-SKF or TSK-11 scores, and their relevance is unclear. No previous study has detailed the pattern of change in PROM scores, such as IKDC-SKF, TSK-11, and ACL-RSI scores from pre-ACLR to post-ACLR. The clinician’s ability to understand psychological factors and how they relate to physical factors during the recovery period before and after ACLR is essential to addressing these factors and their potential impact on RTS [1].

Therefore, this study aimed to determine the characteristics of changes in IKDC-SKF, TSK-11, and ACL-RSI scores from pre-ACLR to 6 months post-ACLR. We hypothesized the following: (1) participants’ mean scores would improve significantly from pre-ACLR to 6 months post-ACLR; (2) the proportions of participants whose TSK-11 and ACL-RSI scores remain unchanged or decline from pre-ACLR to 6 months post-ACLR are greater than the proportion of those whose IKDC-SKF scores remain unchanged or decline; (3) there is no association between changes in IKDC-SKF scores and those in TSK-11 and ACL-RSI scores from pre-ACLR to 6 months post-ACLR.

## Methods

### Study design and participation

This was a longitudinal observational study. Participants who underwent primary ACLR between September 2018 and April 2020 were included in this study if they met the following criteria: (1) aged 16–45 years at the time of surgery and (2) participated in sports with a modified Tegner activity scale score [5] of ≥ 5 before ACL injury. Participants were excluded if they met the following criteria: (1) underwent surgery other than ACLR 6 months before reconstruction, (2) underwent multiple ligament reconstruction and lateral extra-articular tenodesis, (3) had a cartilage injury requiring surgery, (4) underwent ACLR previously, (5) had difficulty in visiting the clinic due to distance or social reasons, or (6) had missing questionnaire data.

### Procedures

Demographic information, including age, sex, body mass index the day before ACLR, type of sport played at the time of injury, activity level, participation level, date of injury, date of surgery, and type of injury, were obtained from the patients’ medical records. The type of sport was categorized as collision, contact, limited contact, noncontact, and fixed-object high-impact rotational landing based on a previous study [20]. Activity level was determined using the modified Tegner activity scale [5]. Participation level was categorized as recreation, competitive, and elite, based on a previous study [2]. The types of injury were categorized as contact, indirect contact, noncontact, and others [30, 31]. Ligament endpoints were graded as firm or absent using the Lachman test [21]. Subjective knee function and psychological questionnaires were administered the day before surgery and at approximately 6 months (maximum of 2 weeks before and after) post-ACLR. Ethical approval was obtained from the Ethics Committee (approval no. M2019-019–1). All participants provided written informed consent before participation in the study.

### Surgical technique and postoperative rehabilitation

All procedures were performed by orthopedic surgeons specializing in knee joint surgery. Semitendinosus, semitendinosus and gracilis, or bone-patellar tendon-bone autografts, were used. Participants did not undergo preoperative structured rehabilitation. Their postoperative rehabilitation protocol was based on those used in previous studies [23]. Sports participation was allowed when the following were achieved: at least 6 months had passed after ACLR, the limb symmetry index (LSI) for the single-leg hop test exceeded 90%, and the LSIs of isokinetic extension and flexion torque measured using an isokinetic dynamometer (BIODEX System 4, BIODEX Medical Inc., Shirley, NY, USA) at 60°/s and 180°/s were > 85%. All participants followed the same rehabilitation protocol. However, participants who underwent repair of the middle-posterior segment of the meniscus were prohibited from performing deep squatting to > 90° until 3 months after ACLR.

### Subjective knee function

Subjective knee function was measured using the Japanese version of the IKDC-SKF [10]. The IKDC-SKF comprises items related to knee symptoms, function, and ability to engage in various levels of sports activities and can be used in patients with various types of knee problems [11]. It consists of 19 items, with scores ranging from 0 to 100. Higher scores indicate fewer symptoms, better function, and the ability to perform higher-level sports activity.

### Psychological variables

The Japanese versions of the TSK-11 [13] and ACL-RSI [9] were administered to assess kinesiophobia and psychological readiness to RTS, respectively. The TSK-11 is an 11-item questionnaire, with each item graded on a four-point Likert scale. The total score ranges from 11 to 44, with higher scores indicating greater kinesiophobia. The ACL-RSI is a 12-item questionnaire comprising items related to emotions, confidence in performance, and risk appraisal. The score for each domain is summed and averaged to obtain a total score between 0 and 100, with higher scores indicating greater psychological readiness to RTS [32].

### Data and statistical analyses

The representative value of each score, amount of change, and percentage change from pre-ACLR to 6 months post-ACLR were calculated. The percentage change in the scores was calculated using the following formula:

$$\textit{Parcentage change }(\%)=\frac{\textit{score at 6 months postoperatively-preoperative score}}{\textit{preoperative score}}\times100$$

A positive change was defined as an increase for IKDC-SKF and ACL-RSI scores and a decrease in TSK-11 score. We determined the normality of the distribution of each variable using a histogram and the Shapiro–Wilk normality test. To test the first hypothesis, we analyzed the change in each score from pre-ACLR to 6 months post-ACLR using a paired t-test. Effect sizes (Cohen’s *d*) were calculated for each variable. Cohen’s *d* was used to classify effects as small (0.20–0.50), medium (0.50–0.80), or large (> 0.8) [16]. To test the second hypothesis, the distribution of the percentage change in each score was examined from pre-ACLR to 6 months post-ACLR using a histogram. To test the third hypothesis, correlations of the percentage change of TSK-11 and ACL-RSI scores with those of IKDC-SKF scores were analyzed from pre-ACLR to 6 months post-ACLR using Pearson’s correlation coefficient and Spearman’s rank correlation coefficient. The a priori α level was set at 0.05. Data were analyzed using the SPSS ver. 27.0 (IBM Corp., Armonk, NY, USA) software.

An a priori sample size calculation was conducted using G* Power software 3.1.9.4 [4]. Based on a previous study [8, 26–28] that analyzed changes in IKDC-SKF, TSK-11, and ACL-RSI scores from pre-ACLR to 6 months post-ACLR, the minimum number of participants required was 25 (two-tailed; effect size, 0.76; α error, 0.05; power, 0.95). Power was set to 0.95 to consider the effect of type II errors.

## Results

**Fig. 1:**
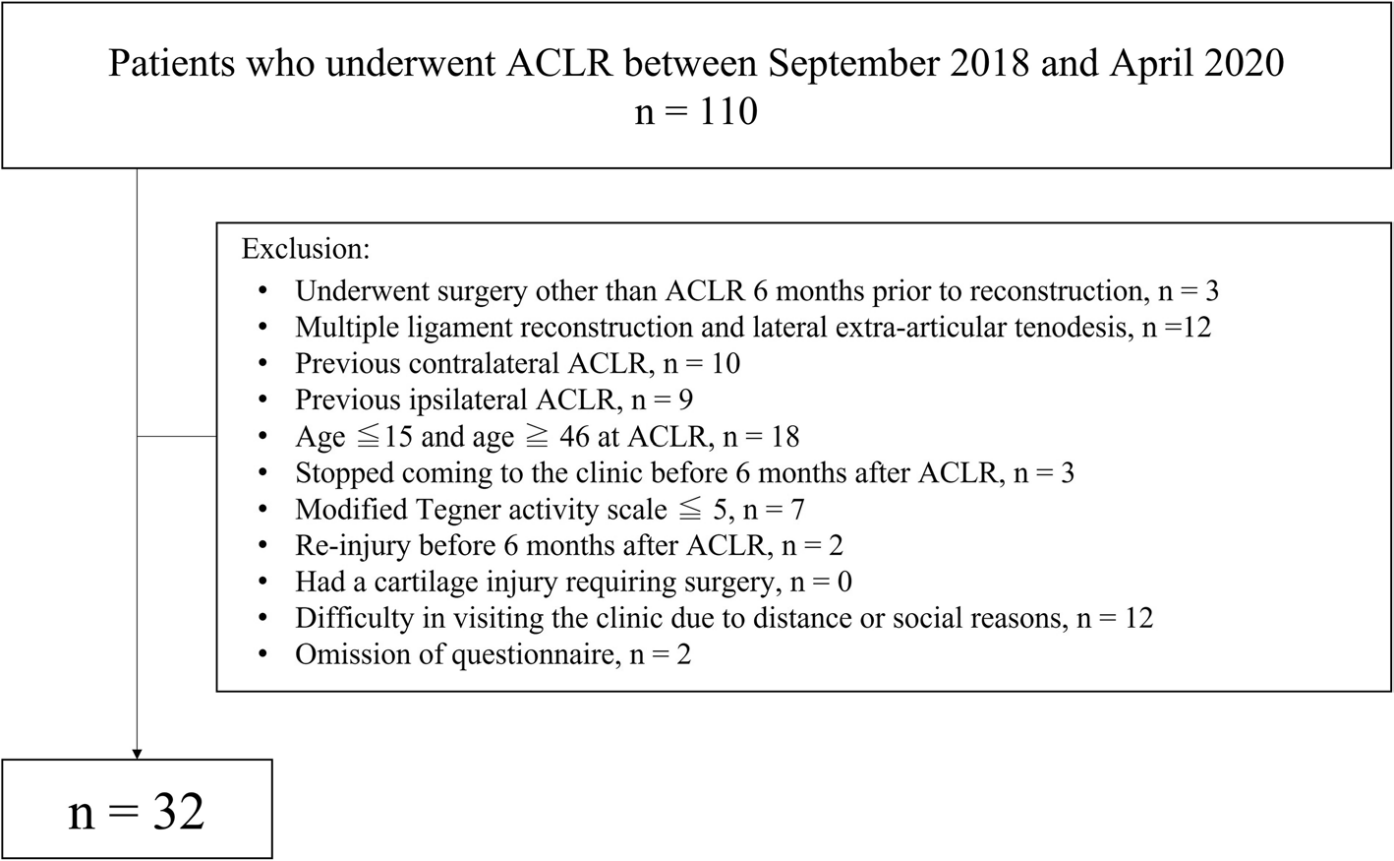
Flow chart of participant enrollment. ACLR, anterior cruciate ligament reconstruction

Thirty-two participants were included in the analysis (Fig. 1). Their demographic information are presented in Table 1. None of the study participants returned to sport at the same competitive level as before the injury at 6 months post-ACLR. Changes in scores of each outcome measure are summarized in Table 2. The IKDC-SKF and ACL-RSI scores significantly increased, whereas the TSK-11 scores significantly decreased from pre-ACLR to 6 months post-ACLR.

**Table 1:** Participants’ demographic characteristics

Age (years)^a^	20.0 (5.0)
Sex (female/male), n	14/18
Body mass index^a^	23.4 (5.2)
Days from injury to surgery^a^	64.0 (67.0)
Type of injury (contact/indirect contact/noncontact/other), n	6/9/15/2
Graft type (ST/STG/BTB), n	25/3/4
Preoperative Lachman test (absent/firm/not graded due to fear), n	24/3/5
Meniscus repair (yes/no), n	16/16
Meniscus resection (yes/no), n	27/5
Preinjury modified Tegner activity scale^a^	8.0 (2.0)
Participation level (recreation/competitive/elite), n	5/18/9
Type of sport (collision/contact/limited contact/noncontact/fixed-object high-impact rotational landing), n	6/15/9/1/1

*ST* Semitendinosus, *STG* ST and gracilis, *BTB* Bone-patellar tendon-bone

^a^Median (interquartile range)

**Table 2:** Comparison of subjective knee function and psychological variables

	Pre-ACLR	Six months post-ACLR	*P* value	Cohen’s *d*	Amount of change	Percentage change (%)
IKDC-SKF	68.5 ± 10.1	82.1 ± 10.2	< .001	1.38	13.6 ± 9.9	21.5 ± 18.7
TSK-11	26.0 ± 3.6	23.6 ± 4.4	.003	0.39	–2.4 ± 4.2	–8.7 ± 15.2
ACL-RSI	58.1 ± 17.9	64.4 ± 17.6	.018	0.34	6.2 ± 14.1	6.1 (46.2) ^a^

Mean ± standard deviation, ^a^median (interquartile range)

*ACLR* Anterior cruciate ligament reconstruction, *IKDC-SKF* International Knee Documentation Committee Subjective Knee Form, *TSK-11* Tampa Scale for Kinesiophobia-11, *ACL-RSI* Anterior Cruciate Ligament-Return to Sport after Injury scale

**Fig. 2:**
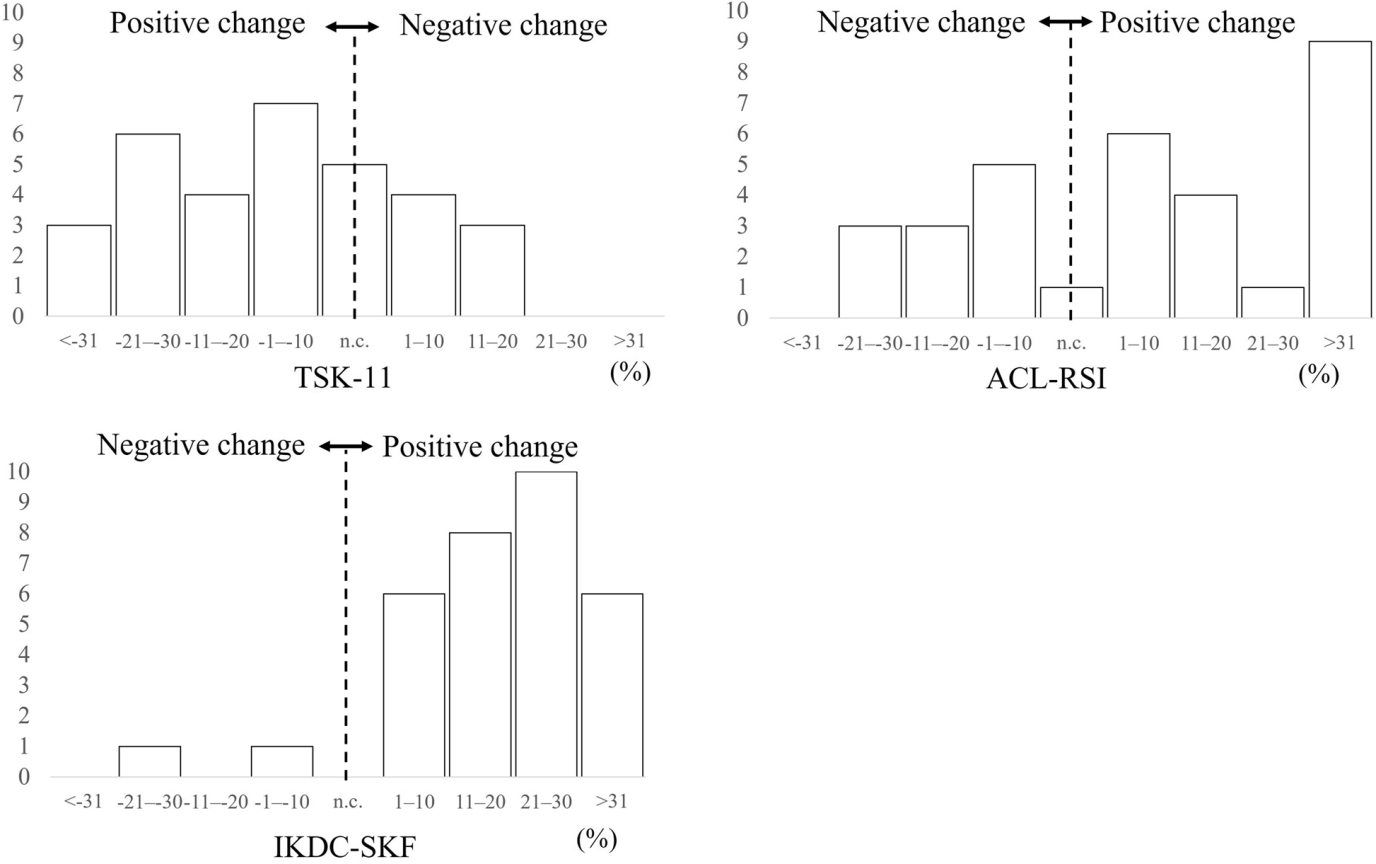
Percentage changes in the scores from pre-ACLR to 6 months post-ACLR. ACLR, anterior cruciate ligament reconstruction; TSK-11, Tampa Scale for Kinesiophobia-11, ACL-RSI: Anterior Cruciate Ligament Return to Sport after Injury scale; IKDC-SKF, International Knee Documentation Committee Subjective Knee Form; n.c., no change. The x-axis shows the change in the score from pre-ACLR to 6 months post-ACLR. The y-axis shows the frequency. A positive change was defined as an increase in IKDC-SKF and ACL-RSI scores and a decrease in TSK-11 score

Histograms of the percentage change in IKDC-SKF, TSK-11, and ACL-RSI scores from pre-ACLR to 6 months post-ACLR are shown in Fig. 2. The IKDC-SKF scores of two participants (6.3%) changed negatively and the TSK-11 of 12 participants (38.0%) and ACL-RSI scores of 12 participants (38.0%) remained unchanged or changed negatively from pre-ACLR to 6 months post-ACLR.

**Fig. 3:**
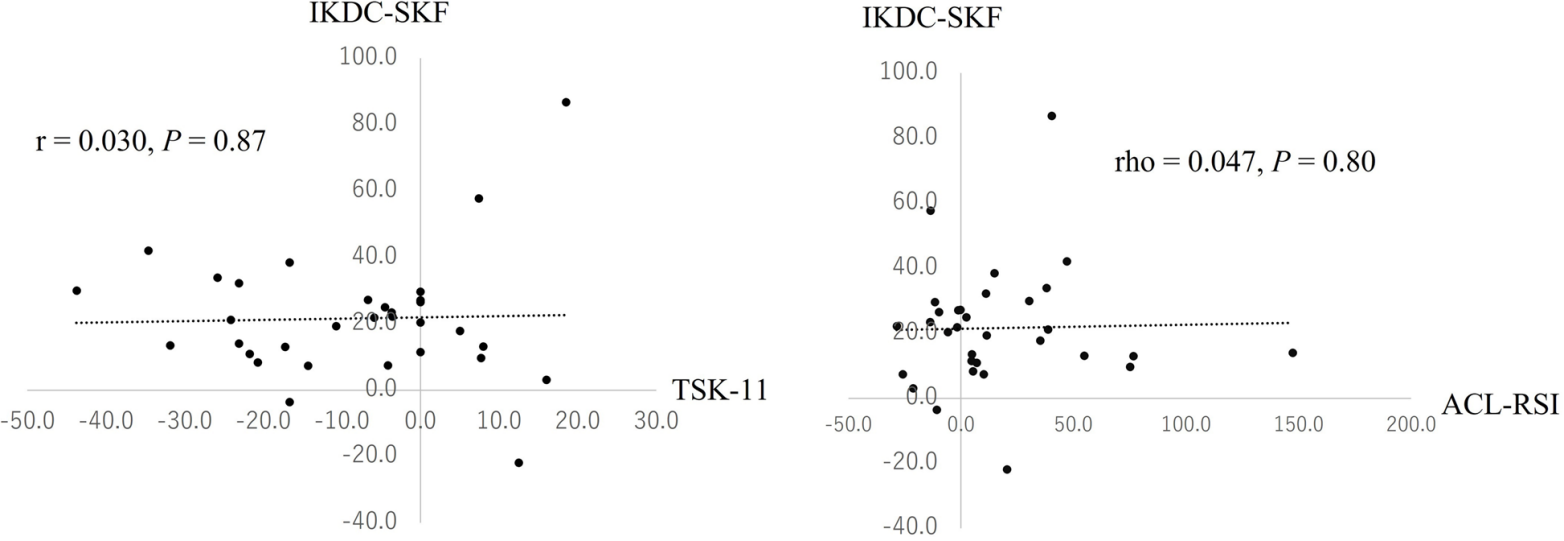
Correlations of percentage change in the IKDC-SKF score from pre-ACLR to 6 months post-ACLR with that in the TSK-11 and ACL-RSI scores. ACLR, anterior cruciate ligament reconstruction; IKDC-SKF, International Knee Documentation Committee Subjective Knee Form; TSK-11, Tampa Scale for Kinesiophobia-11; ACL-RSI, Anterior Cruciate Ligament Return to Sport after Injury scale

The results of the correlation analyses are shown in Figs. 3. The percentage change in the IKDC-SKF score from pre-ACLR to 6 months post-ACLR was not significantly correlated with that in either the ACL-RSI or TSK-11 scores.

## Discussion

The main finding of this study was that subjective knee function and psychological state showed variable patterns of change from pre-ACLR to 6 months post-ACLR. The results of this study support our hypotheses.

In this study, participants’ IKDC-SKF scores significantly changed positively from pre-ACLR to 6 months post-ACLR with a large effect size (*d* = 1.38). This is consistent with the results of previous studies that have analyzed changes in the IKDC-SKF scores (Table 3) [15, 17, 26]. In patients who underwent ACLR, the minimal clinically important difference (MCID) for the IKDC-SKF score at 6 months postoperation was 10.7 points [10]. This indicates that the subjective knee function of this study’s participants significantly changed positively after ACLR and subsequent rehabilitation.

**Table 3:** Reference values from previous research

Outcome	Author	Patient characteristics	Score		Calculated effect size (*d*)
			Pre-ACLR	Six months post-ACLR	
IKDC-SKF	*Raoul, *et al*.* [26]	-	62.0 ± 14.3	76.1 ± 11.9	1.07^b^
	*Magnitskaya, *et al*.* [17]	-	65 (54–77)^a^	80 (68–89)^a^	-^b^
TSK-11	*Hartigan, *et al*.* [8]	Non-coper	26.0 ± 6.5	15.6 ± 3.4	2.01^b^
		Coper	22.6 ± 5.5	17.8 ± 4.8	0.93^b^
	*Rhim, *et al*.* [27]	Intervention	25.9 ± 3.4	24.5 ± 3.9	0.38
		Placebo	24.6 ± 7.2	20.7 ± 6.3	0.58
		Control	25.0 ± 4.5	23.3 ± 5.5	0.34
ACL-RSI	*Raoul, *et al*.* [26]	-	40.2 ± 22.1	60.2 ± 20.9	0.93^b^
	*Sadeqi, *et al*.* [28]	-	41.3 ± 25.4	58.3 ± 22.3	0.71^b^
	*Rhim, *et al*.* [27]	Intervention	52.3 ± 11.7	59.0 ± 13.6	0.53
		Placebo	50.9 + 23.2	62.6 ± 27.8	0.46
		Control	60.8 ± 16.0	66.3 ± 11.6	0.39

Mean ± standard deviation, ^a^median (interquartile range), ^b^*P* < .05

*ACLR* Anterior cruciate ligament reconstruction, *IKDC-SKF* International Knee Documentation Committee Subjective Knee Form, *TSK-11* Tampa Scale for Kinesiophobia-11, *ACL-RSI* Anterior Cruciate Ligament-Return to Sport after Injury scale

In this study, TSK-11 scores significantly decreased (changed positively) from pre-ACLR to 6 months post-ACLR; however, the effect size was small (*d* = 0.39). In a previous study [27] that examined the effect of video intervention on the psychological state of patients after ACLR, TSK-11 scores did not decrease significantly from pre-ACLR to 6 months post-ACLR in either the intervention or control groups and the effect size was similar to that in the present study (Table 3). In a cohort study in which patients with ACL injury were grouped as copers (excellent dynamic knee stability) or non-copers (poor dynamic knee stability), both groups showed a significant decrease in TSK-11 scores from pre-ACLR to 6 months post-ACLR, with a large effect size [8]. In a previous study [8] on patients with an ACL injury who underwent preoperative structured rehabilitation to improve quadriceps muscle strength symmetry and other functions, TSK-11 scores were significantly decreased following intervention. A previous study by Rhim et al. [27] in which TSK-11 was not reduced by video intervention provided no description of preoperative rehabilitation. Similarly, in this study, no structured preoperative rehabilitation was provided. These differences in preoperative interventions may have been influenced the results.

The ACL-RSI scores increased significantly from pre-ACLR to 6 months post-ACLR. This is consistent with the results of previous cohort studies [26, 28] that have analyzed changes in the ACL-RSI scores. However, the effect size (*d* = 0.34) in our study was smaller than that in previous studies (Table 3). A previous study [27] reported that although the ACL-RSI score did not improve significantly from pre-ACLR to 6 months post-ACLR, the preoperative score was higher than that reported in other studies [26, 28]. Therefore, the difference in effect size may be due to the difference in preoperative scores. However, compared to the preoperative ACL-RSI scores of other previous study (mean: 52.5) [19], only the data of this study are not extremely high.

Interestingly, the proportion of patients whose score did not change positively from pre-ACLR to 6 months post-ACLR was higher for TSK-11 (38%) and ACL-RSI (38.0%) than for IKDC-SKF (6.3%) scores. In addition, the percentage change in IKDC-SKF scores was not associated with that in the TSK-11 and ACL-RSI scores (Fig. 3). These results indicate that while the subjective knee function score changed positively from pre-ACLR to 6 months post-ACLR, the psychological state of a large percentage of the participants did not change positively. Although the TSK-11 and ACL-RSI scores significantly changed positively from pre-ACLR to 6 months post-ACLR (Table 2), this overall change may have been influenced by some participants who showed great change. The association of the percentage change in IKDC-SKF scores and the scores of psychological measures has not been analyzed in previous studies. Since some cross-sectional studies have identified significant correlations between psychological scales such as the TSK-11, ACL-RSI, and subjective knee function such as IKDC-SKF, it is believed that there is a relationship between knee function and psychological status [6, 28]. However, based on the results of this study, changes in subjective knee function and psychological status from pre-ACLR to 6 months post-ACLR may not be interdependent. Changes in subjective knee function and psychological status from pre-ACLR to 6 months post-ACLR should be assessed separately.

### Clinical implications

If trainers, therapists, and clinicians only focus on the knee function of patients undergoing ACLR, athletes may participate in sports with a negative psychological state despite improvement in knee function. In such cases, athletes will participate in sports with a lack of psychological readiness to RTS and fear of reinjury, leading to increased rates of reinjury and failure to RTS at the preinjury level [14, 18, 25]. Based on the study results, it may be necessary to evaluate not only the knee function but also the psychological state of each patient before surgery and observe the changes in them [29]. Patients with positive changes in physical function but negative changes in psychological state scores may need to consider delaying RTS [12]. It may also be necessary to provide psychological interventions, such as guided imagery and relaxation in collaboration with psychological specialists [3].

### Study limitations

This study had some limitations. First, this study evaluated only two-time points: preoperation and 6 months postoperation. Therefore, changes in each score in the other intervals are unknown. Second, although the minimum sample size to test the current study hypothesis was met, multivariate analysis including covariates, such as sex, age, and competition level was not performed. Third, the individual characteristics of patients who showed worsened scores were unknown. Fourth, there may have been events that affected their psychology postoperatively; however, these factors are unknown. Fifth, the MCID from pre-ACLR to 6 months post-ACLR for the TSK-11 and ACL-RSI scores has not been determined. Therefore, it is impossible to weigh the improvements in scores. Sixth, the change in each score from pre-ACLR to 6 months post-ACLR may be influenced by the baseline (pre-ACLR) score. Therefore, further data accumulation is needed to strengthen the evidence of this study. Finally, none of the patients were allowed to RTS before evaluation. The scores could be altered by the patients’ participation in sports. In the future, a long-term cohort study may be able to address these limitations. In addition, evaluating outcomes such as RTS and reinjury would provide more clinically meaningful data.

## Conclusions

IKDC-SKF, ACL-RSI, and TSK-11 scores changed significantly positively from pre-ACLR to 6 months post-ACLR. However, the proportion of participants whose scores did not change positively from pre-ACLR to 6 months post-ACLR was higher for TSK-11 (38.0%) and ACL-RSI (38.0%) than for IKDC-SKF (6.3%). No significant correlation was observed between subjective knee function and psychological state scores regarding the percentage change from pre-ACLR to 6 months post-ACLR. Therefore, changes in subjective knee function and psychological status from pre-ACLR to 6 months post-ACLR should be assessed separately.

## Data Availability

Datasets generated or analyzed during this study are not publicly available due to privacy concerns. However, they are available from the corresponding author upon reasonable request.

## Abbreviations

ACLR: Anterior cruciate ligament reconstruction
ACL-RSI: Anterior Cruciate Ligament Return to Sport after Injury scale
IKDC-SKF: International Knee Documentation Committee Subjective Knee Form
LSI: Limb symmetry index
MCID: Minimal clinically important difference
PROMs: Patient-reported outcome measures
RTS: Return to sport
